# Multiplex PCR assay for the simultaneous identification of race specific and non-specific leaf resistance genes in wheat (*Triticum aestivum* L.)

**DOI:** 10.1007/s13353-022-00745-5

**Published:** 2022-12-29

**Authors:** Aleksandra Noweiska, Roksana Bobrowska, Julia Spychała, Agnieszka Tomkowiak, Michał T. Kwiatek

**Affiliations:** Department of Genetics and Plant Breeding, Faculty of Agronomy, Horticulture and Bioengineering, Poznań University of Life Sciences, 11 Dojazd Str, 60-632 Poznań, Poland

**Keywords:** Molecular markers, Multiplex PCR, Leaf rust resistance, Slow rusting genes, Wheat

## Abstract

Race-nonspecific resistance is a key to sustainable management of pathogens in bread wheat (*Triticum aestivum* L.) breeding. It is more durable compared to race-specific immunity, conferred by the major genes (R), which are often overcome by pathogens. The accumulation of the genes, which provide the resistance to a specific race of a pathogen, together with the introduction of race-non-specific resistance genes is the most effective strategy aimed at preventing the breakdown of genetically conditioned immunity. PCR markers improved the productivity and accuracy of classical plant breeding by means of marker*-*assisted selection (MAS). Multiplexing assays provide increased throughput, reduced reaction cost, and conservation of limited sample material, which are beneficial for breeding purposes. Here, we described the process of customizing multiplex PCR assay for the simultaneous identification of the major leaf rust resistance genes *Lr19*, *Lr24*, *Lr26*, and *Lr38*, as well as the slow rusting, race-nonspecific resistance genes: *Lr34* and *Lr68*, in thirteen combinations. The adaptation of PCR markers for multiplex assays relied on: (1) selection of primers with an appropriate length; (2) selection of common annealing/extension temperature for given primers; and (3) PCR mixture modifications consisting of increased concentration of primers for the scanty band signals or decreased concentration of primers for the strong bands. These multiplex PCR protocols can be integrated into a marker-assisted selection of the leaf rust-resistant wheat genotypes.

## Introduction

Leaf rust, caused by the causal fungus, *Puccinia triticina* Eriks. (syn. *P. recondita* Rob. ex Desmaz. f. sp. *tritici*), is one of the most common and damaging diseases of wheat worldwide (Huerta-Espino et al. 2011). Under optimal environmental conditions, the infection can significantly reduce the kernel weight and the number of kernels per ear, which reflects in the yield reduction, even up to 70% (Kolmer 1996; Chen et al. 2013).

More than 80 *Lr* (leaf rust) genes, 83 *Yr* (yellow rust) genes, and 63 *Sr* (stem rust) genes have been identified and referred in the Catalogue of Gene Symbols for Wheat (McIntosch 2019). Most of them belong to the group of major resistance genes (R genes) (Kolmer et al. 2008a, b). Several effective *Lr* resistance genes were successfully introduced into wheat from related species, such as: *Thinopyrum elongatum* (syn. *Agropyron elongatum*), *Th. intermedium* and *Secale cereale* (Cai et al. 2001, Zhang et al. 2010, Salina et al. 2015). The *Lr19* gene was transferred into the wheat genetic background from *Th. elongatum* and affects the plant hypersensitivity response and increases the grain yield (Gupta et al. 2006). *Th. elongatum* is also a source of the *Lr24* gene, which provides leaf resistance at seedling and adult plant stages (Rai et al. 2021; Schachermayr et al. 1995). Another *Thinopyrum* species, *Th. intermedium*, is a source of *Lr38* gene, which ensures a stable resistance of seedlings and adult plants to many isolates of *P. triticina*, which appeared in Europe. Another wheat relative, rye (*Secele cereale* L.), is a donor of the *Lr26* gene, which is present in many varieties of wheat carrying 1RS.1BL chromosome translocation (Mesterházy et al. 2000; Mebrate et al. 2008; Salina et al. 2015).

Another type of resistance genes, called race-nonspecific or horizontal resistance genes, provide the durable resistance against all races of different pathogens (Ellis et al. 2014). In case of leaf rust, this type of resistance is manifested by the slow progression of the infection (Caldwell et al. 1968). So far, eight “slow rusting” genes have been identified in the wheat genome: *Lr34 (*= *Yr18/Sr57/Pm38)*, *Lr46 (*= *Yr29/Sr58/Pm39)*, *Lr67 (*= *Yr46/Sr55/Pm46)*, *Lr68*, *Lr74*, *Lr75*, *Lr77*, and *Lr78* (Suenaga et al. 2003, Singh et al. 1998, Hiebert et al. 2010, Herrera-Foessel et al. 2012, McIntosh et al. 2015, Singla et al. 2017, Kolmer et al. 2018a, b). The *Lr34* is the best known and characterized “slow rusting” loci (Dyck et al. 1987), which confers a partial resistance to leaf rust, as well as a moderate level of resistance to the stripe rust, caused by *Puccinia striiformis* (*Yr18*; McIntosh 1992, Singh 1992); powdery mildew, caused by *Blumeria graminis* (*Pm38;* Spielmeyer et al. 2005); stem rust, caused *Puccinia graminis* (*Sr57*; Dyck 1992); and barley yellow dwarf virus (*Bdv1*, Singh 1993). The presence of the *Lr34* gene is also manifested by the appearance of leaf tip necrosis in certain environments, which can be used as a phenotypic assay for the presence of the gene (Dyck 1991; Singh 1992). The *Lr34* gene encodes a pleiotropic ATP-binding cassette (ABC) transporter, of the ABCG subfamily (Krattinger et al. 2009). Resistant and susceptible haplotypes can be distinguished by three single nucleotide polymorphisms (SNP) (Lagudah et al. 2009). Other slow rusting genes, *Lr46* and *Lr68*, were described in Pavon and Parula cultivars, respectively (Herrera-Foessel et al. 2012). Both genes showed a smaller effect than *Lr34*, but the combined effect of *Lr34*, *Lr46*, and *Lr68* can assure near immunity (Martinez et al. 2001). The effects of these genes when appearing alone, are moderate; however, they can be used as backbone genes in combinations and interactions with other major genes, resulting in high levels of durable resistance.

The selection of individuals is an important stage in wheat breeding. Currently, the classic selection is supported by the identification of molecular markers related to valuable traits. Although molecular techniques are highly specific, sensitive, and reliable, nevertheless they are expensive, laborious, and time-consuming. Multiplex polymerase chain reaction (multiplex PCR) since its first description in 1988 (Chamberlain et al. 1988) has been successfully utilized in many areas of DNA testing. It allows the simultaneous amplification of two or more *loci* in one reaction (Henegariu et al. 1997a), which reduces the time and costs of investigation; hence, it has been applied in marker-assisted selection in breeding programs.

The aim of this work was to develop the multiplex PCR protocols for the simultaneous identification of the major leaf rust resistance genes *Lr19*, *Lr24*, *Lr26*, and *Lr38* together with the “slow rusting” genes *Lr34* and *Lr68* in various combinations, which can be used for the selection process in wheat breeding.

## Materials and methods

### Plant material and DNA isolation

The experimental material consisted of 8 accessions of spring wheat (Table 1), which were reported as sources of leaf rust resistance genes, including four near-isogenic lines of ‘Thatcher’ with single genes (*Lr 24*, *Lr26*, *Lr34*, *Lr38*); one spring wheat cv. ‘Chinese Spring’ which was used as negative control for *Lr19*, *Lr24*, *Lr26*, and *Lr38* and one spring wheat cv. ‘Artigas’ representing negative control for *Lr34* and *Lr68*. The plant material was obtained from the National Small Grains Germplasm Facility, National Small Grains Collection in Aberdeen, Idaho, USA. Seeds were plated for germination on Petri dishes. GeneMATRIX Plant & Fungi DNA Purification Kit (EURx Ltd., Poland) was used for DNA extraction from the leaf tissue of 10-day-old seedlings. Leaf tissue samples were finely ground in liquid nitrogen and the remaining tissue structures were subsequently solubilized by lysis in the presence of a special buffer, which preserves the integrity and stimulates quantitative recovery of all traces of DNA. Further, proteinase K was used to digest contaminating proteins. “Sol-P” buffer and ethanol were added to provide selective conditions for DNA binding during brief centrifugation, while contaminants pass through the resin in the spin column. Traces of contaminants remaining on the resin were removed in two wash steps. The final DNA concentration after diluting the samples with Tris buffer (EURx Ltd., Poland) was 50 ng μl^–1^.

**Table 1:** Presence of molecular markers in tested wheat (*T. aestivum* L.*)* genotypes

No	Cultivar/genotype	Plant ID	*Xwmc221* for *Lr19*	*Sr24#12* for *Lr24*	*P6MI2* for *Lr26*	*Xwmc773* for *Lr38*	*csLv34* for *Lr34*	*csGs* for *Lr68*
1	Agatha	CItr 14,048	** + **	−	−	** + **	−	−
2	Lr24	GSTR 425	−	** + **	−	−	−	−
3	Lr26	GSTR 427	−	−	** + **	** + **	−	−
4	Lr34	GSTR 433	−	−	−	−	** + **	−
5	Lr38	GSTR 437	−	−	−	** + **	−	−
6	Parula	PI 520,340	−	−	−	−	** + **	** + **
7	CIGM98.745–1	PI614023	** + **	−	** + **	** + **	−	−
8	Glenlea	CItr 17,272	−	−	−	−	** + **	** + **

### Molecular markers and PCR reactions

In order to develop freely available multiplex PCR protocols, we have used molecular markers, as well as primer sets, which are accessible and were already published. The molecular markers sequences, sizes of amplified products, and references are presented in Table 2.

**Table 2:** Primer sequences and reaction details for PCR reactions with leaf rust resistance markers according to literature

Gene — marker	Primers sequences	Annealing temperature*	Product amplified	Sources
*Lr19 — Xwmc221*	F: ACGATAATGCAGCGGGGAAT R: GCTGGGATCAAGGGATCAAT	61 °C	200 bp ( +) 220 bp ( −)	Gupta i in. 2006
*Lr24 — Sr24#12*	F: CACCCGTGACATGCTCGTAR: AACAGGAAATGAGCAACGATGT	65 °C	500 bp ( +)	Mago et al. 2005a
*Lr26 — P6M12*	F: GTACTAGTATCCAGAGGTCACAAG R: CAGACAAACAGAGTACGGGC	57 °C	260/360 bp ( +)	Mago et al. 2005b
*Lr38 — Xwmc773*	F: GAGGCTTGCATGTGCTTGA R: GCCAACTGCAACCGGTACTCT	61 °C	140/160 bp ( +)	Mebrate et al. 2008
*Lr34 — csLv34*	F: GTTGGTTAAGACTGG TGA TGG R: TGCTTGCTATTGCTGAATAGT	55 °C	150 bp ( +) 230 bp ( −)	Lagudah et al. 2006
*Lr68 — csGs*	F: AAG ATT GTT CAC AGA TCC ATG TCA R: GAG TAT TCC GGC TCA AAA AGG	60 °C	385 bp ( +)	Herrera-Fossel et al. 2012

^*^Recommended annealing temperature according to the literature

The initial PCR reaction was performed using FastGene® OptimaHotStart ReadyMix (NIPPON, Germany) according to the manufacturer’s protocol. PCR mixture consisted of 1.25 µg of template DNA; PCR-grade H_2_O, 1 × FastGene® OptimaHotStart ReadyMix (NIPPON, Germany), and 1-µM primers (Sigma-Aldrich, USA). PCR was performed using a Labcycler thermal cycler (SensoQuest GmbH). The PCR reaction was performed with the following cycling protocol: (a) initial denaturation of 3 min at 95 °C; (b) 35 cycles of denaturation of 30 s at 95 °C, primer annealing of 30 s at 5 °C lower than the calculated melting temperature (Tm) of the given primer set; and elongation step of 1 min at 72 °C; and (c) a final elongation step of 7 min at 72 °C. The initial conditions of the PCR reaction were later developed by modifications of (1) primers’ concentrations and (2) annealing temperatures, which were crucial to design functional multiplex PCR protocols.

The PCR amplification products were electrophoresed in 2% agarose (Bioshop, Canada Inc.) gel in 1 × TBE buffer (Bioshop, Canada Inc.) stained with 4 µl of Midori Green Advanced DNA Stain (NipponGenetics Europe, Germany) per 100 ml and photographed under UV light in a Molecular Imager Gel Doc™ XR UV system with the Biorad Bio Image™ Software.

## Results

The optimization of the PCR method for multiplex PCR reaction design consisted of three phases: (1) selection of effective, available molecular markers for *Lr19*, *Lr24*, *Lr26*, *Lr34*, *Lr38*, and *Lr68*, whose amplicons’ sizes allow to distinguish particular alleles in the multiplex assay; (2) adjustment of common annealing temperatures using gradient PCR; and (3) primers’ concentrations manipulation to obtain easy-to-interpret banding pattern on the gel.

### Selection of markers

All markers available in the literature were analyzed. Multiplex PCR assays involve a large number of primers; hence it is required that the designed primer should be of appropriate length. Here, primers of short length, in the range of 18–22 bases were used. Primer sets, that yield amplicons with the appropriate product sizes, which can be easily distinguished using standard agarose gel electrophoresis have been selected (Table 2).

### Annealing temperature

**Fig. 1:**
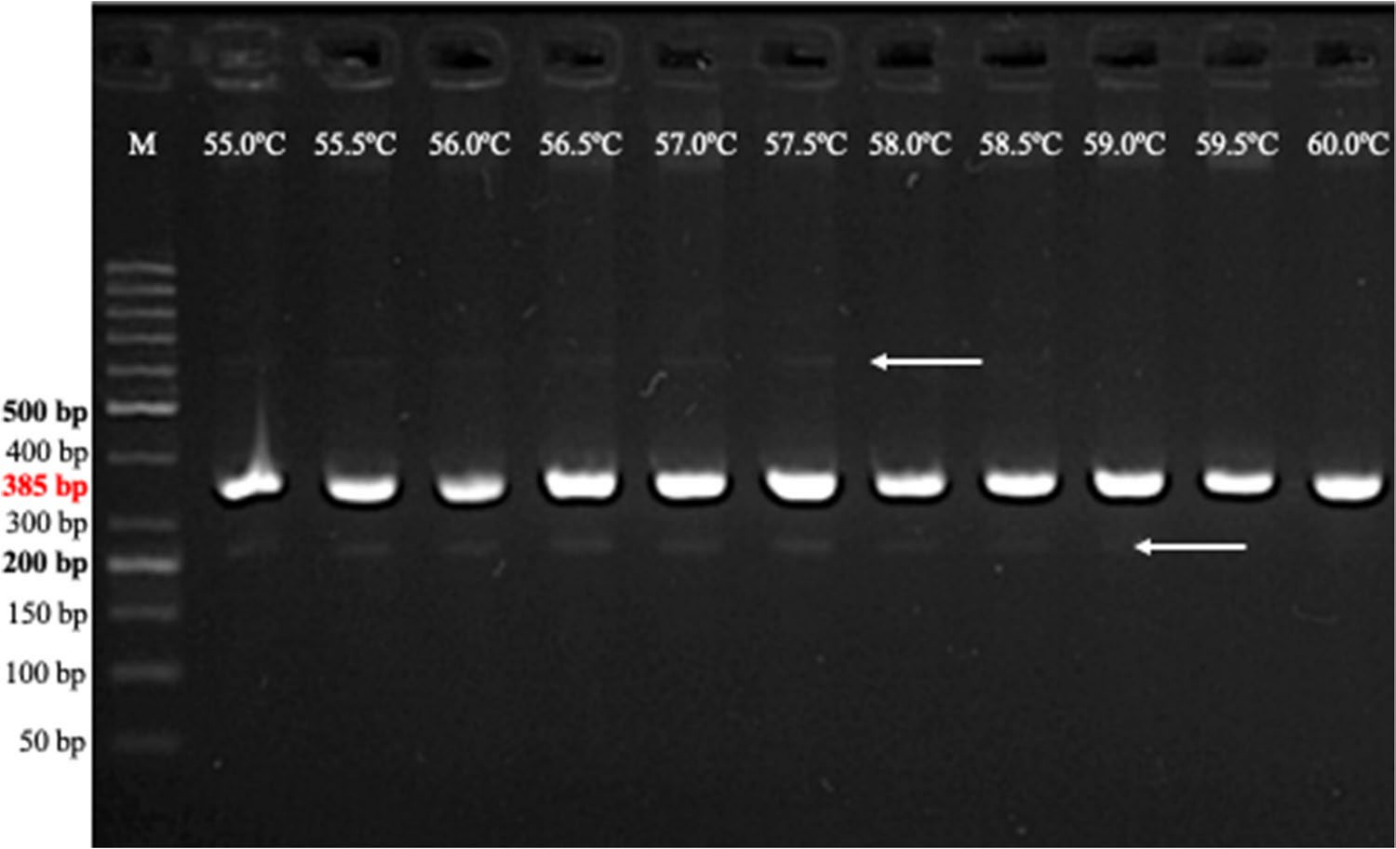
Gradient of annealing temperature (55–60 °C) for *csGs* marker linked to *Lr68* gene. Arrows indicate the unspecific products, which were eliminated by the modification of primers concentrations. GeneRuler 50-bp DNA Ladder was used as DNA standard/ladder used to compare the various banding patterns

The next step was to analyze the interactions between primer sets in the course of multiplex PCR reaction. Primers with similar melting temperatures (*T*_m_), preferably between 55 and 65 °C were used (Table 2). Hence, further approaches were conducted in order to test the suitable annealing/extension temperatures by thermal gradient (Fig. 1). A Tm variation of between 5 and 10 °C was acceptable for primers used in a pool; hence, we have used the lowest annealing temperature (55 °C) for all combinations (Table. 3). However, lower annealing temperatures yielded some unspecific products (Fig. 1), which were eliminated by the modification of primers concentrations in the following step. The common temperature for annealing allowed to multiplex thirteen marker combinations (Table 3), which could be used according to the need of the experiment or breeding program.

**Table 3:** Reaction conditions for developed combinations of multiplex PCR

No	Combination	Concentrations [μM] of each pair of primers	Annealing temperature
C1	*Lr19* + *Lr34*	0.8 + 0.8	55 °C
C2	*Lr24* + *Lr34*	0.8 + 0.8	
C3	*Lr19* + *Lr24* + *Lr34*	0.96 + 0.48 + 0.96	
C4	*Lr24* + *Lr34* + *Lr68*	0.56 + 1.04 µl + 0.8	
C5	*Lr19* + *Lr34* + *Lr68*	0.67 + 0.67 + 0.67	
C6	*Lr19* + *Lr24* + *Lr34* + *Lr68*	0.67 + 0.4 + 1.12 + 0.67	
C7	*Lr19* + *Lr26*	0.8 + 0.8	
C8	*Lr24* + *Lr26*	0.8 + 0.8	
C9	*Lr19* + *Lr24* + *Lr26*	0.8 + 0.8 + 0.8	
C10	*Lr19* + *Lr26* + *Lr38*	0.8 + 0.8 + 0.8	
C11	*Lr24* + *Lr26* + *Lr38*	0.56 + 1.04 + 0.8	
C12	*Lr19* + *Lr24* + *Lr26* + *Lr38*	0.8 + 0.48 + 1.12 + 0.8	
C13	*Lr19* + *Lr24* + *Lr26* + *Lr34* + *Lr68*	0.8 + 0.48 + 1.12 + 1.12 + 0.8	

### Specificity

**Fig. 2:**
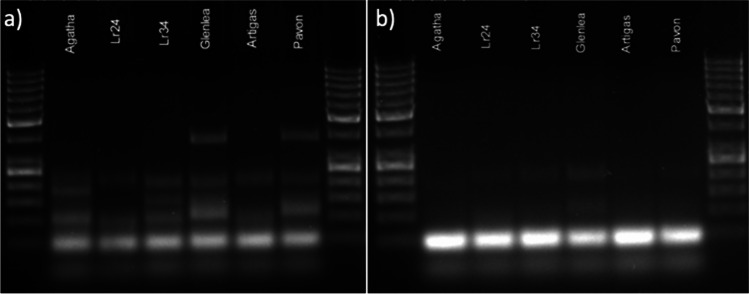
Electropherogram of multiplex PCR reaction for *Xbarc71* (*Lr24*) and *csLv34* (*Lr34*) markers showing **a** false amplicons or primer-amplicon interactions and **b** false amplification due to primer dimers. GeneRuler 50 bp DNA Ladder was used as DNA standard/ladder used to compare the various banding patterns

**Fig. 3:**
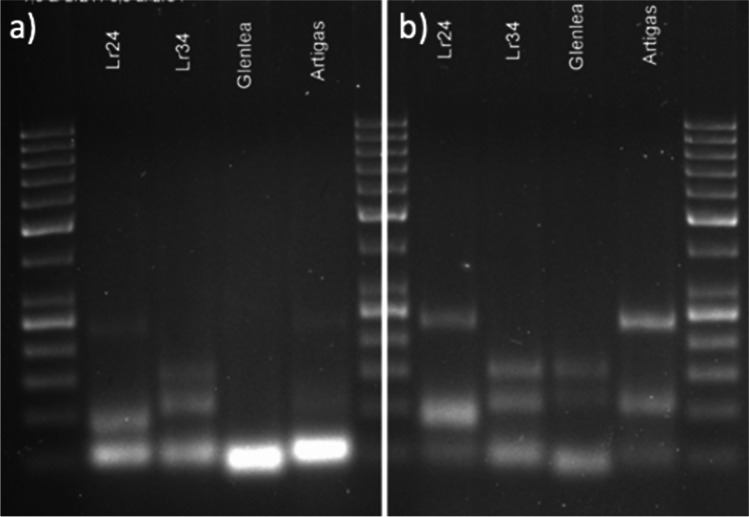
Modification of the concentration of primers for *Xbarc71* (*Lr24*) and *csLv34* (*Lr34*). Initial primers concentrations of **a** 1 μM each and **b** 0.8 μM each

**Fig. 4:**
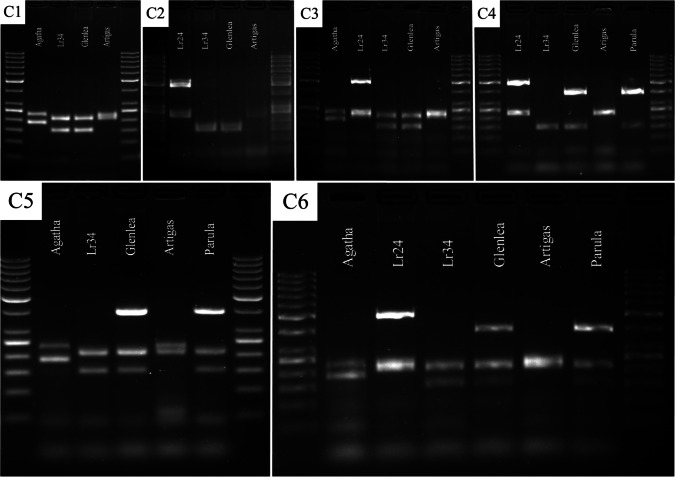
Electropherogram showing the presence of markers: *Xwmc221* (for *Lr19*), *Sr24#12 (Lr24)*, *csLv34 (Lr34)*, and *csGs (Lr68*) in wheat varieties in various combinations (C1–C6)

**Fig. 5:**
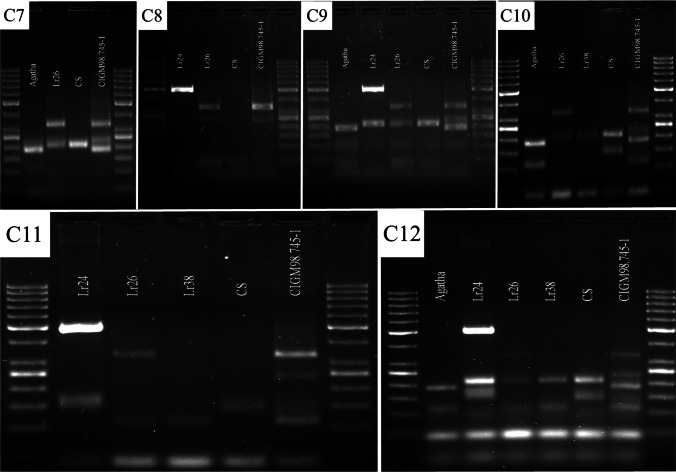
Electropherogram showing the presence of markers: *Xwmc221* (for *Lr19*), *Sr24#12 (Lr24)*, *P6MI2 (Lr26)*, and *Xwmc773 (Lr38)* in wheat varieties in various combinations (C7–C12)

**Fig. 6:**
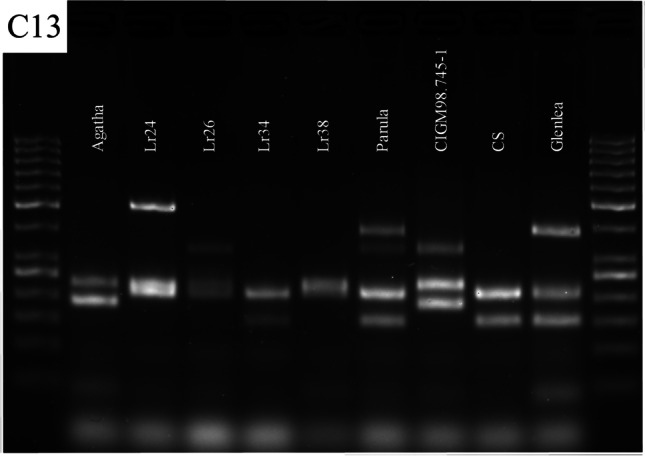
Electropherogram showing the presence of markers: *Xwmc221* (for *Lr19*), *Sr24#12 (Lr24)*, *P6MI2 (Lr26)*, *csLv34 (Lr34)*, and *csGs (Lr68*) in wheat varieties in C13 combination

It is important to consider the specificity of primers to the target sequences, while preparing a multiplex assay, especially since competition exists when multiple target sequences are in a single reaction vessel. At first, the multiplex reactions were performed by adding primers in equimolar concentrations. Initially, equimolar primer concentrations of 1 μM each were used in the multiplex PCR. The results suggested that individual primer concentrations need further modifications. In the case of *Lr24* loci, the multiplex assay adjustment process was initiated with the *Xbarc71* marker that yields 103- or 85-bp products, linked with resistant or susceptible alleles, respectively (Table 2). However, the *Xbarc71* primers together with primers of other markers provided false amplicons or primer-amplicon interactions (Fig. 2a) or false amplification due to primer dimers (Fig. 2b). All approaches failed, so another marker, *Sr24#12*, was selected for *Lr24* loci identification. However, PCRs with more than two sets of primers resulted in uneven amplification, with some barely visible products or even absent (Fig. 3). To overcome these obstacles, we performed a set of PCR experiments with different proportions of primers in the reaction. The final concentration of the primers ranged between 0.4 and 1.5 μM. Generally, the PCR mixture modification consisted of increased concentration of primers for the scanty band signals or decreased concentration of primers for the strong bands (Fig. 3a, b; Table 3). The abovementioned alternations of PCR protocol allowed to perform easy-to-interpret results of multiplex PCR reactions for thirteen combinations of primers (Table 3, Figs. 4, 5, and 6).

## Discussion

In this study, we have demonstrated a set of thirteen multiplex PCR marker combinations, which can be deployed in the process of marker-assisted selection. We have developed the multiplex PCR protocols for the most effective major genes (*Lr19*, *Lr24*, *Lr26*, and *Lr38*) and slow rusting genes, including *Lr34* and *Lr68*, which are securing the durable resistance of wheat.

Optimalization of the multiplex PCR is a challenging procedure, which is based mainly on a trial-and-error approach. In the literature, only few publications discuss the process of multiplex PCR protocol development (Chamberlain and Chamberlain 1994; Edwards and Gibbs 1994; Henegariu et al., 2018).

An initial solution to difficulties encountered in the development of multiplex PCR has been the use of hot start PCR (Chou et al. 1992). This type of enzyme eliminates nonspecific reactions (particularly production of primer dimers) caused by primer annealing at low temperatures (4 to 25 °C) before commencement of thermocycling (Kebelmann-Betzing et al., 1998). Hence, in our study, we have used the OptimaHotStart polymerase, which is activated only if the reaction mixture is heated at approximately 95 °C for 10 min (the first denaturation step).

First cycles have a substantial effect on the overall sensitivity and specificity of PCR. The success of specific amplification relies on the primers annealing to their target and the rate at which annealed primers are extended along the desired sequence. Optimal annealing depends on primer length and GC content and their concentrations, as well as annealing temperature (Chamberlain & Chamberlain 1994). Thus, the majority of modifications to improve PCR performance have been directed towards the factors affecting annealing and/or extension rates. The optimization of multiplex PCRs can raise several obstacles including poor sensitivity or specificity and/or preferential amplification of certain specific targets (Polz and Cavanaugh 1998). The presence of more than one primer pair in the multiplex PCR increases the chance of obtaining spurious amplification products, primarily because of the formation of primer dimers (Brownie et al. 1997). In the case of our experiment with *Lr24* and *Lr34* markers, these nonspecific products were amplified more efficiently than the desired target, consuming reaction components and producing impaired rates of annealing and extension. Thus, the optimization of multiplex PCR should aim to minimize or reduce such nonspecific interactions. This may be achieved through the utilization of primers with nearly identical optimum annealing temperatures (primer length of 18 to 30 bp or more and a GC content of 35 to 60% may prove satisfactory) and should not display significant homology either internally or to one another (Henegariu et al., 2018). Modifications including primers concentration as well as other PCR components such as PCR, dNTPs, and enzyme concentrations in multiplex PCR over those reported for most uniplex PCRs usually result in modest improvement in the specificity of the assay. Increasing the concentration of these factors may increase the likelihood of mis-priming with subsequent production of spurious nonspecific amplification products. However, optimization of these components in multiplex PCRs that are designed for simultaneous amplification of multiple targets may prove beneficial. For example, in the multiplex PCR for simultaneous detection of wheat and soybean, different ratios of primers concentrations were used for analysis of 21 different commercial food products (Shin et al. 2021).

Multiplex PCR become widely adopted within the plant breeding industry for high-throughput genotyping in a variety of applications, such as germplasm characterization and MAS (Yap et al. 2016), identification of genetically modified organisms (Bak and Emerson 2019) and pathology testing (Otti et al. 2016). It is a quick method that also allows to lower research costs and shortens the duration of the experiment. For example, Froidmont (1998) used multiplex PCR to identify 1BL/1RS translocation in wheat, together with the screening of resistance loci for yellow rust (*Yr9*), stem rust (*Sr31*), leaf rust (*Lr26*), and powdery mildew (*Pm8*). Moreover, Sumiková and Hanzalová (2010) studied rust leaf resistance genes *Lr26* and *Lr37* and stated that the multiplex PCR method can be a breakthrough tool in identifying varieties resistant to the disease. Tomkowiak et al. (2019) developed a multiplex PCR protocol to accelerate the identification of efficient major leaf rust resistance genes: *Lr11*, *L13*, *Lr16*, and *Lr26* using the following molecular SSR markers: *Xwmc24*, *Xwmc261*, *Xgwm630*, *Xwmc764*, and *P6M12*, respectively. Multiplex PCR assays were also developed for simultaneous screening of slow rusting genes. Skowrońska et al. (2019) published a protocol for joint identification of *Lr34* and *Lr46*, and later, they improved the protocol by adding a molecular marker linked to another slow rusting gene, *Lr68* (Skowrońska et al. 2020). What is more, Lata et al. (2021) optimized a multiplex polymerase chain reaction (PCR) for simultaneous detection of two important leaf rust resistance genes: seedling resistance gene *Lr24* and slow rusting gene *Lr68.*

In conclusion, in this study, we optimized and developed 13 combinations of multiplex PCR conditions for simultaneous identification of markers linked to both effective race-specific (*Lr19*, *Lr24*, *Lr26*, and *Lr38*) and durable, non-specific leaf resistance genes (*Lr34* and *Lr68*). These protocols can be used to accelerate the marker-assisted resistance breeding of common wheat, which meet the recent expectations of the breeders.
